# Yield of Echocardiography with Bubble Studies Among Acute Ischemic Stroke Patients

**DOI:** 10.3390/jcm13216555

**Published:** 2024-10-31

**Authors:** Jonathan Hu, Anson Yoong-Chee Lee, Kazuma Nakagawa, Kimberly Vu, Zia Rehman Khan, Michael Tanoue

**Affiliations:** 1 John A. Burns School of Medicine, University of Hawaii, Honolulu, HI 96813, USA; 2 The Queens Medical Center, Honolulu, HI 96813, USA

**Keywords:** acute ischemic stroke, cryptogenic stroke, patent foramen ovale, bubble study, healthcare waste

## Abstract

**Background:** Transthoracic (TTE) and transesophageal (TEE) echocardiographic studies with agitated saline, also known as “bubble studies” (BSs), are used to diagnose patent foramen ovales (PFOs) in cryptogenic strokes (CSs). Guidelines limit PFO closure recommendations to CS patients ≤ 60 but BSs are often performed as part of standard order sets, leading to inappropriate studies in older patients with already-established stroke etiologies. **Methods:** This retrospective single-center study included acute ischemic stroke patients between January 2021 and June 2022 and assessed the stroke etiology and number of the echocardiographic studies performed. **Results:** A total of 663 patients were admitted during this period with 413 (62.3%) classified as non-CS. Overall, 390 (58.8%) TTE and 40 (6.0%) TEE with BSs were conducted. Of that, non-CS patients received 252 TTE and 17 TEE with BSs. PFOs were diagnosed in 36 CS and 18 non-CS patients and 14 of the 15 PFO closures were performed in the CS patients for primary stroke prevention. The one closure in the non-CS patient was performed in conjunction with the open excision of a mitral valve mass. **Conclusions:** Therefore, the majority of the BSs performed in non-CS patients yielded no change in management. Our study identifies a large volume of diagnostic studies that are not supported by current clinical guidelines and instead, may contribute to healthcare waste. A new institutional protocol should be established to improve operational efficiency and reduce the downstream implications of diagnostic overuse in ischemic stroke care.

## 1. Introduction

Healthcare spending in the United States supersedes that of any other country, making up 17.8% of its gross domestic product [1]. Data suggests that approximately 20–30% of this spending is considered “waste” and does not add value to patient care [2]. In addition, overtreatment with “low value” tests contributes to USD 200 billion of healthcare waste annually [3]. Echocardiography, a non-invasive and frequently performed inpatient diagnostic study, exemplifies such tests with increased costs and limited yield in the settings of syncope [4] and angina [5]. More recently, the use of echocardiography in acute ischemic stroke (AIS) workup has become a point of interest. Most AISs are secondary to cardioembolic events, large-artery atherosclerosis, and small-artery diseases, but approximately one-third are cryptogenic or of undetermined origin [6]. A paradoxical embolus through a patent foramen ovale (PFO) is one mechanism of cryptogenic stroke (CS) and may be diagnosed using transthoracic (TTE) or transesophageal (TEE) echocardiogram with agitated saline, also known as a bubble study (BS) [7]. PFOs were previously controversial due to their uncertain association with stroke but recent trials demonstrating the superior prevention of stroke recurrence with PFO closures compared to antiplatelet therapy have revitalized interest in PFO detection strategies [8,9,10,11,12]. Current guidelines recommend PFO closure only in CS patients aged ≤60 years old, with consideration up to 65 in select low-risk patients with no other stroke etiologies [13]. Despite this, TTE or TEE with a BS are often part of AIS workup and are automatically ordered regardless of patient age or established AIS cause. This practice has obvious negative downstream consequences including the use of limited resources, increased healthcare costs, and potential patient harm [14]. This single-center study evaluates the utilization rate of BSs, and its yield in patients diagnosed with AIS who met age criteria for PFO closure.

## 2. Materials and Methods

This was a single-center retrospective, cross-sectional study of AIS patients admitted between 1 January 2021 to 6 January 2022. This study was approved by the local institutional review board and informed consent was not applicable. The inclusion criteria was the diagnosis of AIS by ICD-10: I63 and the exclusion criteria were hemorrhagic stroke, subarachnoid hemorrhage, TIA, stroke from cerebral venous thrombosis, and stroke secondary to procedural/surgical complication. AIS etiology was determined using the TOAST classification (i.e., large-artery atherosclerosis, cardioembolism, small-artery disease, other determined etiology, and cryptogenic) by a board-certified neurologist, based on the available clinical information and neuroimaging data. The primary endpoint was the utilization rate of TTE and TEE with or without a BS. The secondary endpoints were the rates of PFO closure referrals and percutaneous closures performed. To determine the yield of the diagnostic study, the “number needed to diagnose” (NND) was calculated to express the number of diagnostic studies needed to detect a clinically significant PFO, which led to PFO closure (NND = # of PFO closure/# of bubble studies).

## 3. Results

### Demographics and Outcomes

**Figure 1:**
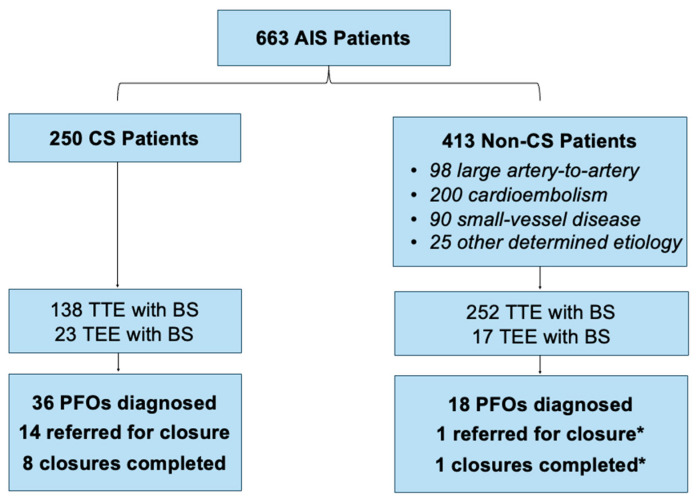
Flow chart demonstrating echocardiogram utilization for AIS etiologies. * Adjunct PFO closure during open excision of a mitral valve mass.

A total of 663 AIS patients [98 (14.8%) large artery-to-artery, 200 (30.2%) cardioembolism, 90 (13.6%) small vessel disease, 25 (3.8%) other determined etiology, 250 (37.7%) cryptogenic] were included in this study. The baseline data are shown in Table 1. Overall, 390 (58.8%) TTE with a BS and 40 (6.0%) TEE with a BS were conducted. PFO was diagnosed in 37 (5.6%) patients using TTE with a BS and 17 (2.4%) patients using TEE with a BS. Ultimately, 15 (2.6%) patients with PFO were referred for closure, and 9 (1.7%) patients underwent PFO closure (see Figure 1). The only PFO closure in the non-CS group was a secondary procedure performed during the open excision of a mitral valve mass, not as a primary measure for CS-related stroke prevention. Consequently, only eight closures were closed with the primary aim of stroke prevention. Based on this, the NND for the entire population was 54. Even among the 200 AIS patients with clear cardioembolic stroke etiology who would not qualify for PFO closure, 109 (54.5%) patients still underwent TTE with a BS.

## 4. Discussion

Recent randomized control trials have renewed interest in PFO diagnostics, after percutaneous PFO closure demonstrated superiority over antiplatelet therapy in preventing recurrent strokes [8,9,10,11,12]. Current guidelines maintain recommendations for percutaneous PFO closure in patients ages 18–60 and in select patients up to 65, with limited evidence for those older [13,15,16]. Furthermore, closure is limited only to CS patients with diagnosed PFOs. Despite this criterion, BSs are often utilized as part of AIS workup for all ages, raising concerns for the overall cost effectiveness and yield of a BS [17]. Our single center study demonstrates that TTE or TEE with a BS is frequently performed for AIS regardless of etiology. Out of our 663 AIS patients, 413 were non-CS. In general, non-CS etiologies would not be candidates for PFO closure as their mechanism of stroke is unrelated to the PFO. Thus, a diagnostic study detecting an incidental PFO may produce minimal change in treatment and ultimately be unnecessary [13]. Echocardiography is a safe, readily available, and cost-effective imaging modality that is often utilized to drive the diagnosis of AIS etiology, and related decision-making with respect to medical therapy [18]. That said, the routine use of echocardiography in the setting of AIS remains controversial, and various guidelines fail to agree on whether echocardiography should be limited to select higher risk patients or discouraged as part of routine management altogether [19,20]. Our findings demonstrate that a disproportionate number of patients with non-CS presentations were referred for additional TTE workup with a BS even though they would have clearly been excluded from PFO closure. Even among CS patients, only 32.8% of those who underwent PFO workup were ≤60 years old, meeting age-specific criteria for closure. Out of the 54 PFOs diagnosed, only 15 underwent closure and, of that, 14 were in the CS group for primary stroke prevention. The closure performed in the non-CS was not intended for stroke prevention and, instead, was an adjunct procedure performed in the setting of an open left mitral valve mass excision. This outcome largely mirrors that of Maggiore et al. who also demonstrated that BSs, while frequently used in routine AIS workup, had minimal subsequent changes in management, as many patients already had readily identifiable risk factors for stroke [21]. Without clear benefit, this practice has many consequences within healthcare utilization and patient safety and should be critically reevaluated.

The cause of this practice pattern may be driven by a combination of various factors. At our institution, AIS patients are often admitted by emergency department providers or Internal Medicine hospitalists who prioritize ruling out immediately actionable pathologies as opposed to determining CS vs. non-CS etiologies. Electronic order sets aid this process and allow them to easily select TTE or TEE with or without a BS. Up to 30% of AIS are of cardioembolic sources unrelated to PFOs and TTE/TEE’s have been shown to guide clinical management in such cases [22,23]. Furthermore, stroke patients are at higher cardiovascular risk, making cardiac imaging essential in secondary stroke management [24]. With seemingly minimal consequences associated with ordering these studies and obvious detriment in missing identifiable cardiac causes of AIS without echocardiography, the volume of studies we observed is unsurprising. Although PFO closure guidelines have recently placed an emphasis on age criteria [13], current stroke management remains equivocal regarding the routine use of BSs [19,20], making it difficult for providers to justify withholding imaging. Since many of these orders are placed before the final stroke etiology is determined, it may also be difficult for providers to discern whether or not cryptogenic sources should be evaluated for. Even so, our results demonstrate no change in the clinical management of non-CS patients who underwent a BS and raise concerns for healthcare waste. With diagnostic radiology studies representing the second-highest driver of hospital spending in AIS, practice patterns surrounding AIS workup must be critically reassessed [25]. While the data collectively underscores the importance of echocardiography in this patient cohort, it also places emphasis on the need for individualized decision-making when pursuing testing, being inclusive when clinical suspicion remains high, and excluding those who are unlikely to benefit.

The benefits of reducing waste in healthcare operations are paramount to their sustainability. Our data review demonstrates a signal for the overutilization of imaging resources in a large academic center without clear benefit. Additionally, the addition of a BS to this practice not only increases entropy within the healthcare system, but also introduces the risk of patient harm via paradoxical air embolism, infection, and bleeding [14]. Based on the anecdotal experiences within our health system, an additional ~15–25 min is spent on average per study to acquire the additional BS portion when accounting for the time needed to have a nurse help with saline agitation, IV administration, and assisting with simulated Valsalva maneuver when patients are unable. Furthermore, TEEs are more invasive, require sedation, and have greater staffing needs related to sedation, recovery, and increased length of stay. By implementing a guideline-driven practice for these studies, health centers may achieve greater operational efficiency by reducing inappropriate study volume and patient harm related to additional and potentially unnecessary testing. However, this study is not without limitations. Its single-center and retrospective design may limit the generalizability of our findings to other tertiary stroke centers and may not reflect the practice patterns of such institutions. There is also limited longitudinal data to evaluate whether patients who underwent PFO closure had less morbidity and mortality. Future studies are needed to confirm the estimations we provided and if a more stringent AIS workup yielded greater benefits, but our findings ultimately highlight a clear area for improvement in clinical practice, identifying the overuse of BSs and providing a foundation to further optimize resource utilization and enhance patient safety in the future.

## 5. Conclusions

In this single-center study, an excessive number of TTE and TEE with a BS were performed for the workup of AIS and, in particular, non-CS etiologies. A guideline-based standardized institutional practice protocol is needed to reduce the amount of unnecessary diagnostic studies in AIS workup.

## Figures and Tables

**Table 1:** Baseline demographics.

	All	CS	Non-CS
N	663	250	413
Age, years	69.3 ± 14.7	67.2 ± 15.1	70.6 ± 14.3
Female	298 (44.9)	113 (45.2)	185 (44.8)
Race			
White	234 (35.3)	84 (33.6)	150 (36.3)
Asian	264 (39.8)	99 (39.6)	165 (40.0)
NHOPI	112 (16.9)	44 (17.6)	68 (16.5)
Unavailable	38 (5.7)	16 (6.4)	22 (5.3)
Hypertension	493 (74.4)	175 (70.0)	318 (77.0)
Diabetes mellitus	267 (40.3)	95 (38.0)	172 (41.6)
Atrial fibrillation/flutter	139 (21.0)	19 (7.6)	120 (29.1)
CAD or prior MI	131 (19.8)	45 (18.0)	86 (20.8)
CHF	95 (14.3)	24 (9.6)	71 (17.2)
Previous stroke/TIA	82 (12.4)	36 (14.4)	46 (11.1)
Smoking	179 (27.0)	58 (23.2)	121 (29.3)
Hyperlipidemia	403 (60.8)	151 (60.4)	252 (61.0)
Obesity (BMI ≥ 30)	152 (24.9)	53 (21.2)	99 (24.0)

All data are shown as N (%) or mean ± SD. NHOPI, Native Hawaiians and other Pacific Islanders; CAD, coronary artery disease; MI, myocardial infarction; CHF, congestive heart failure; TIA, transient ischemic attack.

## Data Availability

Please contact corresponding author for data.

## Abbreviations

AIS: acute ischemic stroke; BS: bubble study; PFO: patent foramen ovale; CS: cryptogenic stroke; TTE: transthoracic echocardiogram; TEE: transesophageal echocardiogram.
